# The effect of viewing-only, reaching, and grasping on size perception in virtual reality

**DOI:** 10.1371/journal.pone.0326377

**Published:** 2025-06-20

**Authors:** Caterina Foglino, Tamara Watson, Niklas Stein, Patrizia Fattori, Annalisa Bosco

**Affiliations:** 1 Italian Institute of Technology, Genoa, Italy; 2 School of Psychology, MARCS Institute for Brain, Behaviour and Development, Western Sydney University, Sydney, New South Wales, Australia; 3 Institute for Psychology, University of Münster, Münster, Germany; 4 Department of Biomedical and Neuromotor Sciences, University of Bologna, Bologna, Italy; 5 Alma Mater Research Institute for Human-Centered Artificial Intelligence (Alma Human AI), University of Bologna, Bologna, Italy; University of Giessen: Justus-Liebig-Universitat Giessen, GERMANY

## Abstract

In virtual environments (VEs), distance perception is often inaccurate but can be improved through active engagement, such as walking. While prior research suggests that action planning and execution can enhance the perception of action-related features, the effects of specific actions on perception in VEs remain unclear. This study investigates how different interactions – viewing-only, reaching, and grasping – affect size perception in Virtual Reality (VR) and whether teleportation (Experiment 1) and smooth locomotion (Experiment 2) influences these effects. Participants approached a virtual object using either teleportation or smooth locomotion and interacted with the target with a virtual hand. They then estimated the target’s size before and after the approach by adjusting the size of a comparison object. Results revealed that size perception improved after interaction across all conditions in both experiments, with viewing-only leading to the most accurate estimations. This suggests that, unlike in real environments, additional manual interaction does not significantly enhance size perception in VR when only visual input is available. Additionally, teleportation was more effective than smooth locomotion for improving size estimations. These findings extend action-based perceptual theories to VR, showing that interaction type and approach method can influence size perception accuracy without tactile feedback. Further, by analysing gaze spatial distribution during the different interaction conditions, this study suggests that specific motor responses combined with movement approaches affect gaze behaviour, offering insights for applied VR settings that prioritize perceptual accuracy.

## Introduction

In recent years, virtual reality (VR) has shown promise for applications that rely on accurate spatial perception, such as training simulations, therapeutic interventions, and immersive experiences. However, challenges remain in achieving accurate distance and size perception in virtual environments (VEs), as users often underestimate spatial properties compared to real-world environments [1]. In the real world, spatial properties are generally perceived more accurately, especially when measured through action-based tasks [2,3]. This discrepancy limits VR’s effectiveness, particularly in tasks requiring precise spatial judgment. To bridge perceptual gaps between virtual and real environments, researchers have used affordance judgments, such as graspability and reachability tasks, to examine size perception accuracy in high-fidelity graphical models. For example, research has found that users perceive objects in virtual spaces to be smaller than they are in reality, although adding stereo viewing can improve perception accuracy [4,5].

A promising approach to improve spatial accuracy involves allowing users to interact directly with the VE and receive visual feedback on their movements [6–8]. Studies indicate that physical movement, rather than merely simulated walking, is crucial for improving distance estimation accuracy. In fact, Waller and Richardson demonstrated that distance judgments in a VE were not significantly improved by interactions wherein participants experienced only a simulation of walking [1]. Instead, they found that physically walking was essential to improve distance estimation even without visual information, highlighting the importance of body-based sensory information in distance perception in VR. Kelly et al. also found that walking through a VE with visual feedback significantly improved distance judgments [9]. This improvement was observed in both walking distance judgments and size-based distance judgments, indicating a rescaling of perceived space. In contrast, the same authors found that interacting with the VE through reaching did not affect distance judgments. These findings suggest that not all types of physical interactions are equally effective at improving distance judgments in VR. Specifically, actions involving physical translation, such as walking have been shown to enhance spatial perception at extrapersonal distances; similarly, interactions within peripersonal space—such as manual reaching and object manipulation—are thought to support spatial understanding by engaging sensorimotor mechanisms.

A relevant aspect that is essential in understanding how humans perceive distances and spatial relationships is *optic flow*—the visual pattern generated by moving objects as one navigates through space. In fact, during forward self-motion, optic flow provides vital directional and amplitude information about one’s movement [10,11]. When moving, the optic flow enables the perception of “heading” [12], where information derived from the environment, like distance and speed, is processed despite potential ambiguities arising from rotational or translational movements [13]. This flow of visual information helps to anchor spatial judgments to stable reference points, a critical process in natural environments and, thus, highly relevant in VR applications that aim to simulate naturalistic experiences. Beyond enhancing distance perception, optic flow influences postural adjustments and can even induce vection—a sensation of self-motion triggered by visual cues alone, proving that humans can derive displacement information purely from optic flow [14,15]. Research demonstrates that such flow-based cues could refine spatial accuracy in VEs, potentially overcoming the perceptual limitations noted in prior studies. Interestingly, studies on vection have shown its potential for anchoring users’ perception of motion, distance, and spatial layout in VR, which can improve navigation and interaction accuracy in virtual spaces. In the field of size perception, optic flow remains a central component, yet its impact in VEs, particularly when coupled with avatars or body-scaled representations, is complex. Variations in body part size within virtual avatars, such as altering virtual foot dimensions, have been shown to influence action judgments (e.g., stepping over gaps) and spatial estimations, demonstrating that users leverage body-scaled cues to adjust spatial judgments in VR [16]. This relationship between visual body representation and environmental scaling hints at the power of body-based cues, even in the absence of complete avatar embodiment, for enhancing size and distance judgments in virtual environments. Beyond visual representation, tactile feedback further influences size perception in VEs. While visuo-haptic integration can enhance user engagement, it may also lead to perceptual distortions, such as overestimations in size perception. Studies, such as those by de Siqueira et al., reveal that haptic-only feedback tends to produce greater overestimations compared to vision-only conditions [17]. This underscores the importance of balancing sensory feedback to ensure that virtual experiences closely align with real-world expectations, reinforcing the interplay between body representation and multimodal sensory inputs in VR.

Building on the principle of action modulation, studies in real-world contexts have shown that the type of action to be planned or executed biases visual perception toward important action-related features [18–21]. For instance, Wykowska et al. showed that the preparation of a grasping movement facilitated the detection of a target that differed in size from other items, while preparing to perform a pointing movement improved the detection of a target that differed in luminance [18,22]. In fact, size-related parameters are fundamental to prepare the grip aperture of the hand to perform a grasping movement [18] and location-related parameters, such as luminance, are fundamental to perform a pointing movement [23,24]. Similarly, Fagioli et al. instructed a group of participants to plan either a grasping or reaching action based on a visual cue and to perform a visual discrimination task while maintaining the planned action [25]. The results showed that participants were better at detecting size deviations after planning a grasping movement, whereas planning a reaching movement facilitated detection of location deviations. Finally, Bosco et al. demonstrated that the perception of two-dimensional object size is influenced not only before, but also after the execution of movement, and particularly more after grasping than reaching [20]. This finding indicates that the perceived size of objects tends to be adjusted based on the type of action performed.

From these studies, it seems that the perception of an object’s features, such as size, can be facilitated only when the planned action requires the elaboration of those properties. These action-based perceptual shifts are further reflected in fixation patterns on objects, with eye movements aligning to optimize spatial awareness based on specific task demands. For instance, when preparing to reach, the eyes initially saccade to the target location, holding fixation until the hand arrives [26]. In grasping tasks, fixations start on the anticipated contact point for the index finger before shifting to the object’s center of mass. Conversely, during passive viewing, fixations naturally settle on the object’s center of gravity [27]. Desanghere & Marotta found a similar shift in fixation locations during both a grasping movement performed toward real objects and a perceptual task in which participants estimated the size of a two-dimesional object [28]. However, the pattern was reversed in the perceptual task: first, participants fixated on the center of mass and subsequently on the upper part of the object. These variations in eye movement patterns reflect distinct perceptual strategies that align with the action intent, suggesting that task-specific eye movements provide insight into how motor preparation influences spatial perception.

Building on this research, the current study investigates how different interactions – viewing-only, reaching and grasping – affect object size perception in VR in one experiment where participants approach the target from a distance by teleportation (Experiment 1) and in another one where participants approach the target by smooth locomotion (Experiment 2). This study leverages prior findings on action-based perceptual shifts by measuring size estimations before and after object interaction. As size is a critical factor in adjusting hand grip aperture during grasping, we hypothesized that grasping interactions would lead to more accurate size judgments compared to reaching, which primes location information [25], and viewing-only, which does not involve physical movement, serving as a baseline condition. Furthermore, by comparing different approach modalities (Experiment 1 and 2), we aim to assess whether optic flow – which offers directional and distance cues that may enhance perceptual accuracy [10,11] – contributes to more accurate size estimations after smooth locomotion compared to teleportation – one of the most used locomotion type in VR [29]. Finally, by examining the variations in fixation locations during interaction and estimation phases, this study aims to clarify whether specific motor responses can induce or not distinct spatial distribution of eye movements as in two-dimensional studies [27].

We expanded this research to VR because virtual environments enable real-time, immersive interactions that simulate naturalistic affordance judgments. VR uniquely incorporates spatial cues like optic flow and visual feedback—factors not replicable on 2D screens—making it ideal for applications where users need to assess object sizes accurately from a distance, such as in training, remote handling, and rehabilitation therapy.

## Experiment 1

### Methods

#### Participants.

A group of sixteen participants took part in the experiment. One participant withdrew from the experiment after experiencing cybersickness. One participant was excluded from the analyses due to an absolute size estimation error that was approximately 2 SD above the group mean. The final sample comprised fifteen participants (Males = 5, range = 20–30 years; mean age = 24.07 years). The participant sample was selected to match that of a previous study by Bosco et al. (2017), which involved a perceptual adjustment task. Eleven participants had no experience with VR, and five reported limited experience (once only). All participants were students or PhD students, were right-handed, had normal or corrected-to-normal vision, normal colour vision and reported no neurological diseases. All participants gave informed consent and were naïve to the purpose of the experiment. The study procedure was approved by the Bioethics Committee of the University of Bologna on 26^th^ of March 2021 with protocol number 76043 and was in accordance with the Ethical standards of the 2013 Declaration of Helsinki.

#### Materials.

**Fig 1:**
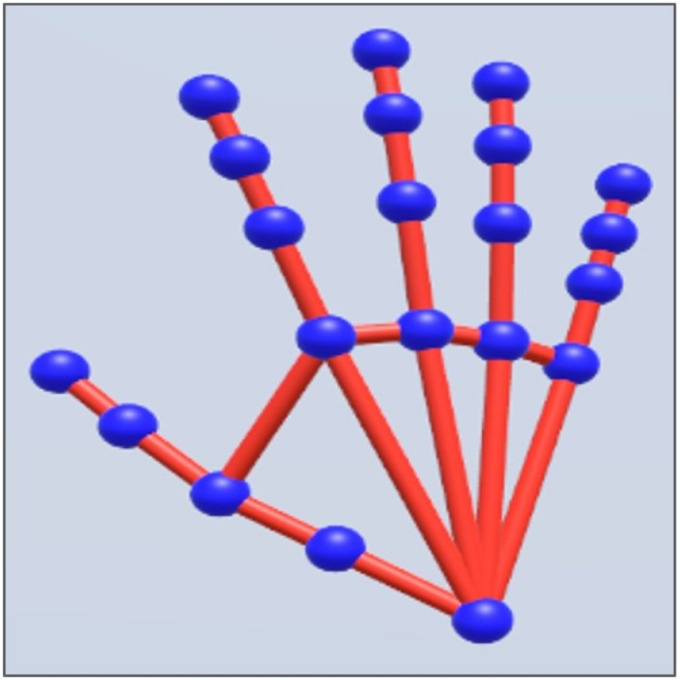
Rendered virtual hand. 21 sphere points (blue spheres) represent the 21 key points of the detected real hand.

The HTC Vive Pro headset was used to display the VE with a resolution of 1440 x 1600 pixels per eye, a refresh rate of 90 Hz and 110 degrees field of view. The Pupil Labs eye tracker add-on (Pupil Labs GmbH, Berlin, using Pupil Capture v. 1.11–4) was used to record eye movements at 90 Hz. One of the HTC Vive controllers was held by participants with the left hand and used to adjust the size of the comparison object during the size estimation phases. Two HTC Vive Base Stations were used to track the location of the headset and the controller during the experimental recordings. The VE was rendered with Unity game engine (Unity Technologies, version 2020.3.23fl) using SteamVR (Valve corporation). Participants were able to visualize a copy of their real right hand via the Vive Hand Tracking software development kit (see Fig 1). This kit provides first-person view hand recognition, based on deep learning algorithms that detect twenty-one key points per hand. These points are then rendered as spherical points in the VE, creating a virtual skeletal representation of the detected hand that mimics the movements of the real one. (Fig 1).

#### Procedure.

**Fig 2:**
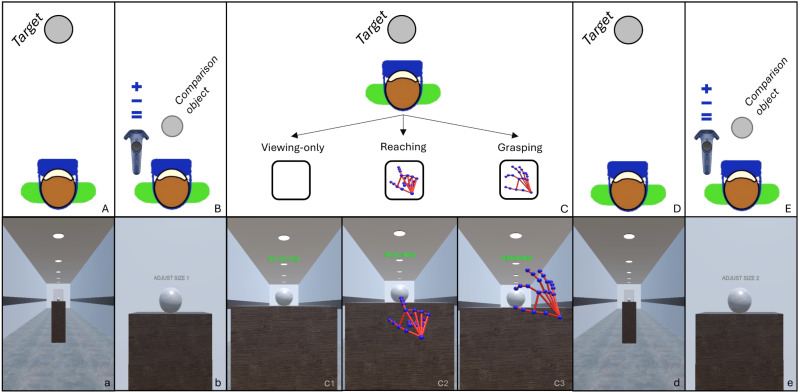
Schematic (first row) and corresponding first-person view (second row) of task sequence of Experiment 1 and 2. A) First target display. Target appears at 3 m distance from the participant. B) First Size Judgment phase. Target disappears and comparison object appears at 0.5 m distance from participant; the controller is used with the left hand to modify the size of the comparison object to match it with the target one. C) Interaction phase. Participant is teleported (Exp. 1) or passively moved (Exp. 2) to stand in front of the target and performs one of the three experimental conditions. The figure does not include the representation of the approach to the target. D) Second target display. Upon successful interaction, participants are automatically transported back at the beginning of the corridor where they see the target from far again. E) Second Size Judgment phase. Same procedure as B). c1) c2) c3) show the text reminder (in green) for each interaction condition.

At the beginning of each experimental session, participants were asked to verbally report their age and their experience with VR technology, then the experimenter gave verbal instructions on the use of the controller and on the task procedure. Participants were asked to estimate the size of a target object by adjusting the size of a comparison one. They were informed they would make this judgment twice: first, after briefly observing the target from afar, and second, after approaching it – via teleportation (Exp. 1) or via smooth locomotion (Exp.2) – and interacting with it using three different movement conditions, which varied by trial (see Fig 2 for a detailed description of the task sequence). Specifically, in the Viewing-only condition, participants were instructed to not perform any movement and wait in front of the target (3 seconds); in the Reaching condition, they were instructed to reach and touch the centre of the target with their right index finger, and in the Grasping condition, they were instructed to first reach the target by extending their arm and hand towards it, and then to grasp it by touching the upper and lower parts of the object with their right index and thumb, respectively. Participants were informed on which action to perform through text prompts at the beginning of each trial and received a text reminder during the interaction phase. The experimenter asked participants to perform the interaction and estimation phases promptly and accurately, and to maintain a standing position throughout the experiment, with the possibility of stretching with minor body movements when needed. Before starting the experimental recording, participants completed a training block (half trials of an experimental block, i.e., 22 trials) to learn the task and become familiar with the virtual environment, allowing them to freely inspect the objects. At the end of the recording session, participants were asked to verbally report the strategy used to estimate the size of the target by the questions detailed in Table 1. This was done to gain insight into the cognitive processes underlying their task performance and to further analyse the effectiveness of the experimental conditions in eliciting specific action-perception interactions. We used a within-subject design, where all participants performed all the experimental conditions in four experimental blocks of forty-five trials each. Between each block, participants had a break and could have a seat until the start of the next experimental block. The entire experimental session including breaks and training phase lasted two hours.

**Table 1:** Questions of the informal interview administered directly after the experiment.

1) Which strategy did you use to give the estimation of target size? (Also: What were you thinking when giving the estimation of the target size?)
2) Did you find any of the interaction conditions more difficult than the others when giving the estimation of the target size? a) If so: can you put the interaction conditions in order of difficulty?

**System calibrations:** At the start of each experimental block, we ran two system calibrations. The first calibration served to match the height of objects in the environment. Fixation point, shelves, and text heights aligned with the headset’s camera height. During this calibration, participants were asked to stand in a relaxed, and natural position with a slight knee bend. This approach allowed to account for slight variations of the participant’s eye level, so that the heights of the target and comparison object, positioned on the shelves, could closely match the participant’s eye level throughout each block. The second calibration was run by the Pupil Labs add-on eye tracker to correlate the eye position with the gaze position in VR.

#### Stimuli.

The experimental VE consisted of a corridor measuring 20 m (length) x 3 m (width) x 3 m (height). The corridor length was set to 20 m to enhance sense of linear perspective. Additional linear perspective cues were added through wall decorations and floor textures. The target object was a grey sphere with five possible diameters: (1) 65 mm, (2) 70 mm, (3) 75 mm, (4) 80 mm, (5) 85 mm. These sizes were selected to represent graspable objects that could accommodate various hand sizes. The comparison object was also a grey sphere, with sizes set at (a) 80%, (b) 100% or (c) 120% of the target size, ensuring that the judgment process was not biased by extreme object dimensions. The target and comparison objects were positioned on a virtual shelf at 0.5 m from the participant during the interaction and size judgment phases, respectively. The target appeared at 3 m when briefly displayed (2 seconds) before the estimation phases (Fig 2, panel A and D) and right before the approach started (not represented in Fig 2). This distance was chosen to provide a natural viewing perspective for inspecting the object from far, while also being long enough to simulate an approach. Only during the interaction phase, a brief auditory feedback was played any time the target was touched, and no haptic feedback was provided. The virtual hand could pass through objects and, consequently, object remained stationary upon contact to ensure consistent behavior and timing across trials. In the size judgment phases (right and left panels of Fig 2), participants adjusted the size of the comparison object using a VR controller to match the target size by increasing, decreasing, or confirming the presented size. The adjustment resolution was randomly set to 0.00045 mm, 0.00050 mm, or 0.00055 mm at the beginning of each trial, and resulted in a continuous change in size with each button press, rather than discrete increments.

### Data analysis

#### Size judgments.

We calculated size estimation errors by subtracting the actual target size from the estimated target size in each judgment phase. We then evaluated the overall difference between size judgments before and after the Interaction phase and tested the effect of the different interaction conditions on the size estimation error. We refer to raw estimation errors as the direct difference between actual and estimated target size where negative values correspond to underestimation of the actual target size and positive values correspond to overestimation of the actual target size. Then, we calculated the absolute estimation errors (i.e., error magnitude) as the unsigned difference between the actual and estimated target size.

To analyse the raw estimation errors between judgments phases and interaction conditions, we used linear mixed models (LMM) with the lmer function (lmerTest package, version 3.1−1 [30]), applying Satterthwaite’s method for p-values and fitting models with restricted maximum likelihood (REML). The results for each predictor in the models are reported as estimated value (β), standard error, t-value, and p-value, with statistical significance set at p < 0.05.

To compare the absolute estimation errors, we used paired t-tests on trial-level data to compare size judgment phases and repeated-measures ANOVAs on participant-aggregated values to compare size judgment phases and repeated-measures ANOVAs to analyse differences between interaction conditions. Post hoc comparisons were adjusted using the Holm-Bonferroni correction. Results include t-value, p-value, mean, standard deviation, and median, with significance at p < 0.05. All the analyses were run using R (version 4.2.1; [31]).

#### Response times.

To explore the impact of different interaction conditions on the estimation phases, we examined the response times associated with reporting the target’s size during the *First* and *Second Size Judgment* phases. Specifically, we measured the time between the moment the comparison object was presented and the instant when either the button to adjust the size or the button to confirm the presented size was pressed. We used repeated-measures ANOVAs to examine differences between size judgments and interaction conditions.

#### Fixations.

To determine whether different patterns of eye movements were elicited depending on the interaction condition, we analysed eye positions during the interaction and estimation phases. We extracted eye data registered with the Pupil Labs add-on for VR at 90 Hz and merged them with the Unity task data. We detected fixations by using a velocity-based algorithm (velocity-threshold identification (I-VT); [32]), setting a gaze velocity threshold of 40° per second and a minimum fixation duration of 100 ms (like in Gao et al. [33]), excluding frames where the quality of the assessment for the eye image from the eye tracker was below the recommended value (0.60) in the Pupil Labs documentation. We analysed the position of the detected fixations on the target during the *Interaction* phase by computing their centroid position [32]. Subsequently, we computed the distance of the centroid of the initial fixation from the centre of the target, and the distance between the centroids of the first and second fixations on the target. The first measure allowed us to assess the position of the initial fixation on the target, while the second measure helped determine whether participants’ gaze remained stable or shifted within the target area during the interaction phase. [27,28]. Additionally, we aimed to investigate whether different interaction conditions would also influence participants’ gaze behaviour when evaluating the target’s size. Therefore, we performed the same analysis for all fixation positions on the comparison object during the estimation phases. We used repeated-measures ANOVAs to examine differences between interaction conditions and size judgments phases.

#### Exclusion criteria.

We identified outlier trials in which participants were too slow during the estimation or interaction phases. This decision was made after observing that participants occasionally exhibited lapses in attention during the estimation phases or experienced delays in the virtual hand display during reaching and grasping. Therefore, to establish acceptable estimation phase durations, we computed the mean and added 3 standard deviations for each size judgment phase. For the interaction phase, we used the mean plus 2 standard deviations. Following these criteria, 4.60% of trials (i.e., 116 trials out of 2520) were excluded from all analyses outlined in the Data analysis section.

### Results

#### Size judgments.

**Judgment phases:** To test whether the accuracy of size estimations would improve after the interaction phase across all conditions (H1), we built a LMM in stages. Our starting point was a baseline model in which *Estimation Error* served as the dependent variable and *Size Judgment Phase* (reference level: *First Size Judgment)* was included as categorical independent variable. Random intercepts for *Participant* and *Experimental Block* were added to account for variability. Subsequently, we expanded the model by adding two additional fixed effects: *Target Size* (i.e., target’s diameter) and the *Scale Factor* (i.e., percentage applied to target’s size to obtain the size of the comparison object). This was done to ensure that the effects of the estimation phases were not depending on specific target sizes presented or were confounded by anchor effects. The ultimate criterion for model selection was the statistical significance of the model comparison using ANOVA (p < 0.001), which demonstrated that the inclusion of *Target Size* and *Scale Factor* significantly improved the model. This key result was supported by converging evidence from lower Akaike Information Criterion (AIC) and Bayesian Information Criterion (BIC) values, as well as a significant chi-square test (see S1 Table). Based on these results, we selected the extended model as the final model (Model 1) was:


$$\begin{array}{c} \begin{array}{c} Model\;1:\;Estimation\;Error\;\sim \;Size\;Judgment\;Phase\;+\;Target\;Size\;+\;Scale\;Factor\;\\ +\;(1|Participant)\;+\;(1|Experimental\;Block) \end{array} \end{array}$$

**Interaction conditions:** To test whether interacting with the object through a grasping movement would result in smaller size estimation errors compared to the other conditions (H2), we built a similar LMM by adding *Condition* as a categorical predictor (reference level: *Grasping*) in interaction with *Size Judgment Phase* (reference level: *Second Size Judgment)*. This model (Model 2) was formulated as follows:


$$\begin{array}{cc} Model\;2:\;Estimation\;Error\; & \sim \;Condition\;*\;Size\;Judgment\;Phase\;+\;Target\;Size\;+\;Scale\;Factor\;\\ & +\;(1|Participant)\;+\;(1|Experimental\;Block) \end{array}$$

**Fig 3:**
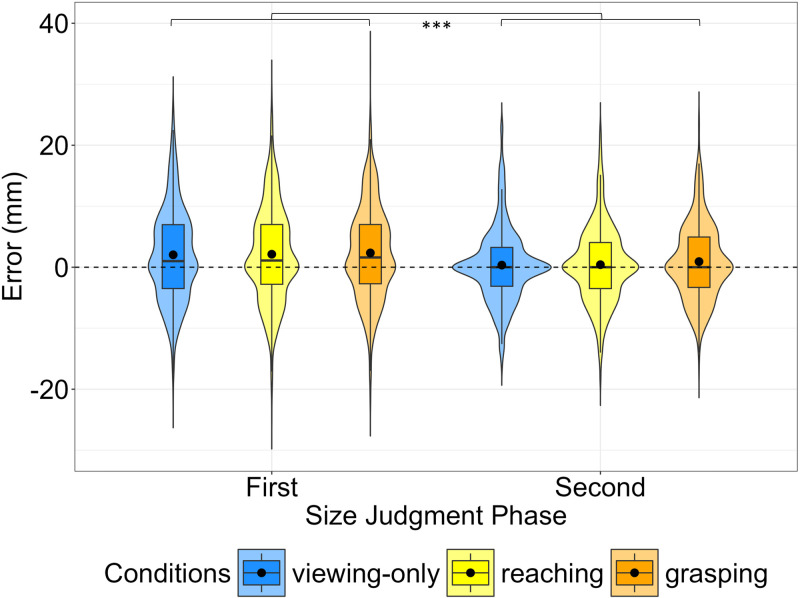
Distributions of the raw size estimation errors of Experiment 1. Violin plots represent the distributions of the errors (raw value) made in the *First* (left violin plots) and *Second* (right violin plots) *Size Judgment* phases*.* Different colours represent the different interaction conditions performed by participants. Box plots are embedded within each violin, showing the median (straight line). The black dot represents the mean for each condition. Positive values (above zero line) indicate overestimation of the target size; negative values (below zero line) indicate underestimation of target size. Asterisks represent significant differences (***p < 0.001).

The overall raw estimation error in the *Second Size Judgment* phase was significantly smaller than in the *First Size Judgment* phase (β = −1.613, SE = 0.146, t = −11.060, p < 0.001, see left and right violin plots of Fig 3), meaning that participants made smaller errors during the *Second Size Judgment* phase (see S2 Table for full model results). In this second phase, the difference in the size estimation between *Viewing-only* and *Grasping* was not significant (p = 0.056), nor was there a significant difference between the size estimation error after *Reaching* and *Grasping* interaction phases (p = 0.089), as it is shown in Fig 3 (right violin plots) (see S3 Table for full results).

We also analysed the absolute values of estimation error to quantify the magnitude of deviation from the actual target size, irrespective of direction. The overall difference between the *First* and the *Second Size Judgment* phases was found to be significant (*t*(2403) = 15.775, p < 0.001) with the *First* phase showing a larger error (Mean = 6.1, Std = 5.1, Median = 5.4) compared to the *Second* one (Mean = 4.5, Std = 4.2, Median = 3.6) suggesting that after the *Interaction* phase, participants were more accurate in the size estimation. The difference in the magnitude of the error between the interaction conditions in the *Second Size Judgment* phase was also found to be significant (*F*(2, 26) = 3.771, p < 0.05). After applying the Holm-Bonferroni correction, a significant difference was found only between *Grasping* and *Viewing-only* (*t*(26) = 2.567, p < 0.05) with larger es*t*imation errors observed in *Grasping* (Mean = 4.7, Std = 4.1, Median = 4.0) compared to *Viewing-only* (Mean = 4.2, Std = 4.3, Median = 3.0). No significant difference was found between *Grasping* and *Reaching* (*t*(26) = 0.482, p = 0.633) or between *Reaching* (Mean = 4.6, Std = 4.2, Median = 3.9) and *Viewing-only* (*t*(26) = −2.115, p = 0.09).

#### Response times.

**Fig 4:**
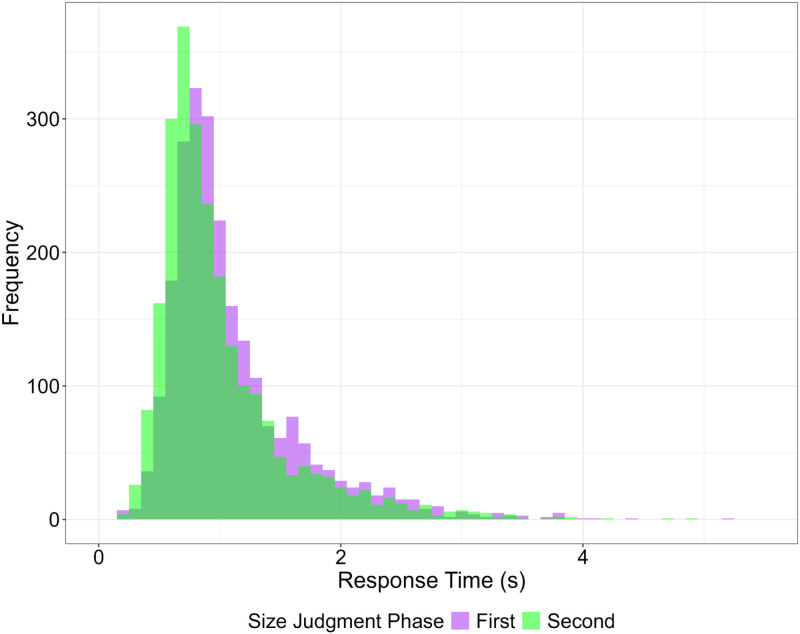
Overlapping histograms of the response times of the *First* (purple histogram) and *Second* (green histogram) *Size Judgment* phases. The plot shows that participants were significantly faster when reporting the size estimation in the *Second Size Judgment* phase.

As it is shown in Fig 4, we observed overall significantly (*F*(1, 4763) = 55.752, p < 0.001) faster response times in the *Second Size Judgment* phase (Mean = 1.00 sec, Std = 0.55, Median = 0.83), compared to the First one (Mean = 1.11 sec, Std = 0.56, Median = 0.94), suggesting that participants were faster in their response after the *Interaction* phase across all conditions. However, we did not find any significant difference between conditions not in the *First* nor in the *Second Size Judgment* phases (*F*(2, 4763) = 0.078, p > 0.05).

#### Fixations.

**Fig 5:**
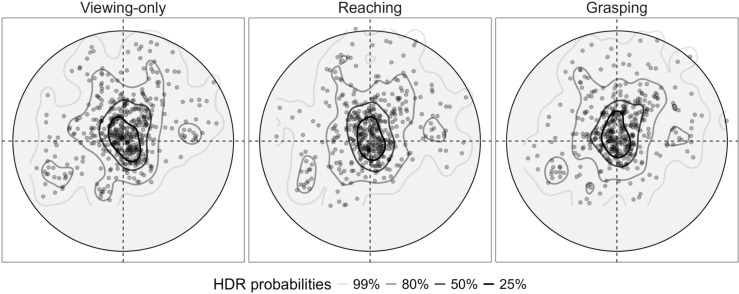
High Density Regions (HDR) of the first fixation made on target for each interaction condition. Each circle represents the 2D view of the sphere-target. The represented diameter of the circles is the average of all five possible diameter values (75 mm) so that the points outside the circles represent fixations on the larger sizes (80 mm and 85 mm). Lines outline different regions which represent different percentages (HDR probabilities) of the total probability distribution of the first fixations made on the target.

The pattern of the initial fixation centroid positions during the Interaction phase is depicted in Fig 5, revealing a consistent pattern within the central 50% of fixations across all conditions. The analysis of fixations patterns on the target during the Interaction phase did not show significant differences between conditions. Specifically, the distance of the first fixation centroids from the centre of the target did not show significant differences (*F*(2, 11) = 0.784, p = 0.481), as well as the distance between the first and the second fixations centroids (*F*(2, 11) = 0.059, p = 0.943). The analysis of the eye positions on the comparison object during the estimation phases revealed an overall significant difference between the *First* and *Second Size Judgment* phases (*F*(1, 5357) = 5.067, p = 0.0244), with the latter showing a shorter distance of the fixations from the centre of the object (Mean = 0.007, Std = 0.006, Median = 0.006), compared to the former one (Mean = 0.008, Std = 0.006, Median = 0.006). However, the interaction between condition and estimation phase was not significant (*F*(2, 5357) = 1.423, p = 0.241).

#### Informal debriefing interview.

The results of the informal interview administered immediately after the experiment (see Table 1) are summarized in Tables 2 and 3 (below). The most frequent strategy reported by participants was trying to recollect the target’s image when adjusting the size of the comparison object during the Second Size Judgment phase (Table 2). Interestingly, when participants were asked to rate the difficulty of the interaction conditions (Table 3) some participants did not report any noticeable distinctions between conditions, while the majority reported Reaching as the most difficult condition. Conversely, Viewing-only was identified as the easiest condition.

**Table 2:** Reported strategies for estimating target size.

Strategy Used	Number of participants	Percentage
Target’s image recollection	9	60%
Combing target’s image recollection and spatial relationships	5	33.33%
Using only other objects as reference	1	6.67%
Total	15	100%

**Table 3:** Summary of participants’ ratings of interaction conditions.

Interaction Condition	Most Difficult	Easiest
Viewing-only	3	6
Reaching	4	2
Grasping	3	2
No noticeable distinctions	5	5
Total	15	15

The table represents the number and the percentage of participants per reported strategy. Most participants recollected the target’s image, while others combined recollecting the target’s image with using the environment spatial relationships, and one used other object in the environment as reference.

The table details the number of participants for each rating. Most participants reported that the Reaching condition was the most difficult to perform, while the Viewing-only condition was the easiest.

## Experiment 2

### Methods

#### Participants.

A group of twenty participants took part in the experiment. Three participants withdrew from the experiment after experiencing cybersickness during the training phase. Seventeen participants were tested (Males = 3, range = 22–34 years; mean age = 28.35 years), two participants were excluded from the analysis because one reported to be influenced by cybersickness, one reported not having understood the task at the end of the recording session. Five participants had no experience with VR, seven reported experiences limited to one occasion only, and three reported a lot of experience with VEs. Participants were compensated with 40$ AUD. The study procedure was approved by Western Sydney University Human Research Ethics Committee (HREC) on 15^th^ of February 2023 with approval number H15296 and were in accordance with the Ethical standards of the 2013 Declaration of Helsinki.

#### Materials.

In this experiment the HTC Vive Pro Eye headset was used. Eye-tracking data were recorded but ultimately not included in the analysis, due to a consistent leftward bias likely caused by a software issue. Despite this bias, the spatial distribution of the eye data appeared realistic and no differences across interaction conditions were found, similarly to Experiment 1. The other materials were the same as Experiment 1.

#### Procedure.

The procedure was the same as in Experiment 1, with the only difference that participants approached the target using smooth locomotion instead of teleportation. This approach provided a continuous optic flow as they moved toward the target, simulating a gradual approach compared to the instantaneous position change in teleportation.

**System calibrations:** The calibration of the objects’ height was the same as Experiment 1. In this experiment, we also performed a third calibration to correct the distance of the virtual hand from the participant’s body. Specifically, we noticed that the distance between the virtual hand and the headset was wrongfully rendered, i.e., the more the hand was extended in space, the more the distance representation was disproportionate. We therefore had to apply a correction to the virtual hand-to-headset distance to match it with the correctly tracked controller-to-headset distance, by performing a linear regression to estimate the best correction factor (r-squared ≥ 0.8). We suspect that the reason for this deviation is a lack of adjustment of the hand tracking algorithm to the camera lenses of the Vive Pro Eye.

#### Stimuli.

The stimuli and task sequence used were the same as Experiment 1 (see Fig 2). The only difference in this experiment was that participants approached the target by attending a linear self-motion simulation (i.e., smooth locomotion), which transported them to the target location at a velocity of 1.2 m/sec – which is considered a normal walking speed [34].

### Data analysis

#### Size judgments.

The same analyses from Experiment 1 for size estimation errors during the *Size Judgment* phases were applied.

#### Exclusion criteria.

Identified outlier trials based on slow duration of estimation or interaction phases were identified with same criteria of Experiment 1. A total of 146 trials (5.40%) were excluded from all the analyses.

### Results

#### Size judgments.

**Fig 6:**
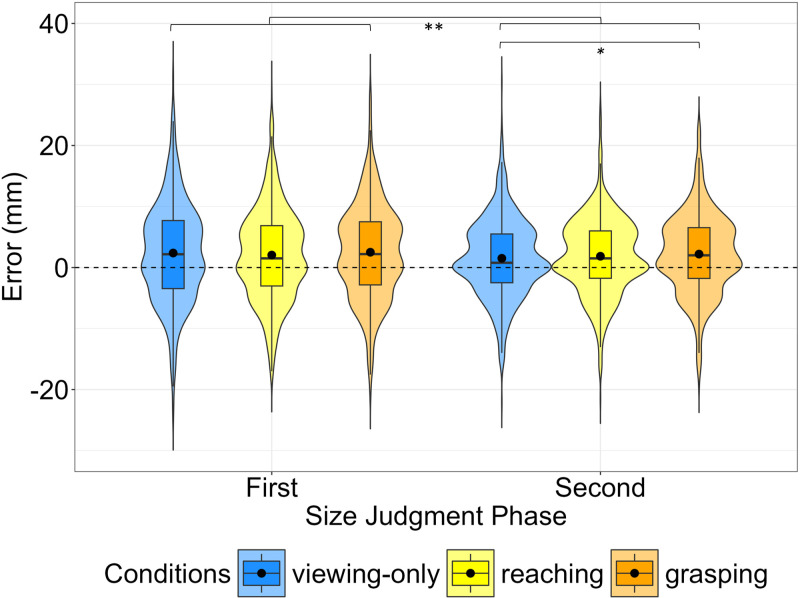
Distributions of the raw size estimation errors of Experiment 2. Violin plots represent the distributions of the raw errors made in the *First* (left violin plots) and *Second* (right violin plots) *Size Judgment* phases. Different colours represent the different interaction conditions. Positive values (above zero line) indicate overestimation of the target size; negative values (below zero line) indicate underestimation of target size. Asterisks represent significant differences (**p < 0.01, *p < 0.05).

The overall difference in the raw error between the *First* and the *Second Size Judgment* phases was found to be significant (β = 0.462, SE = 0.168, t = 2.749, p < 0.01, see left and right violin plots of Fig 6) (see S4 Table for full results). In the *Second Judgment* phase, the raw estimation error was significantly smaller after the *Viewing-only* interaction phase compared to the size estimation error after the *Grasping* interaction phase (β = −0.717, SE = 0.291, t = −2.465, p = < 0.05), whereas we found no significant differences between the size estimation error after the *Grasping* and *Reaching* interaction phases (p = 0.189), as it is shown in Fig 6 (right violin plots) (see S5 Table for full results). The overall difference in the magnitude of the error between the *First* and the *Second Size Judgment* phases was found to be significant (*t*(2553) = 12.031, p < 0.001), with *t*he *First* phase showing a larger error (Mean = 6.3, Std = 5.1, Median = 5.5) compared to the *Second* one (Mean = 5.1, Std = 4.2, Median = 4.5). The difference in the magnitude of the error between the interaction conditions in the *Second Size Judgment* phase was not found to be significant (p = 0.19).

#### Informal debriefing interview.

The results of the informal interview administered immediately after the experiment (see Table 1) are summarized in Tables 4 and 5 (below). Most of participants reported relying on recollecting the target image while adjusting the size of the comparison object during the Second Size Judgment phase.

**Table 4:** Reported strategies for estimating target size.

Strategy Used	Number of participants	Percentage
Target’s image recollection	9	60%
Combing target’s image recollection and spatial relationships	3	20%
Using virtual fingers grip aperture	2	13.33%
Using only other objects as reference	1	6.67%
Total	15	100%

**Table 5:** Summary of participants’ ratings of interaction conditions.

Interaction Condition	Most Difficult	Easiest
Viewing-only	7	6
Reaching	4	7
Grasping	2	0
No noticeable distinctions	2	2

When participants were asked to rate the difficulty of the interaction condition, the majority reported Viewing-only as the most difficult condition. Conversely, Grasping was identified as the easiest condition.

The table represents the number and the percentage of participants per reported strategy. Most participants recollected the target’s image, while others combined recollecting the target’s image with using the environment spatial relationships, two relied on the virtual fingers grip aperture during grasping, and one used other objects in the environment as reference.

The table details the number of participants for each rating. Most participants reported that the Viewing-only condition was the most difficult to perform, while the Grasping condition was the easiest.

### Comparison between size judgments in Experiment 1 and Experiment 2

We performed a comparison analysis between the two experiments to assess the difference in estimation errors, resulting from different means to arrive at the target location. In Experiment 1, participants arrived at target location via teleportation, while in Experiment 2, participants performed the smooth locomotion. We applied a Welch Two Sample t-test to compare the overall raw errors of the Second Size Judgment phase of the two experiments and between each interaction condition. We found an overall significant different raw error value (*t*(4954) = −7.252, p < 0.001) for Experiment 1 (Mean = 0.5, Std = 6.1, Median = 0.0) compared to Experiment 2 (Mean = 1.8, Std = 6.4, Median = 1.5), as well as between each interaction condition of the two experiments (p < 0.001).

**Fig 7:**
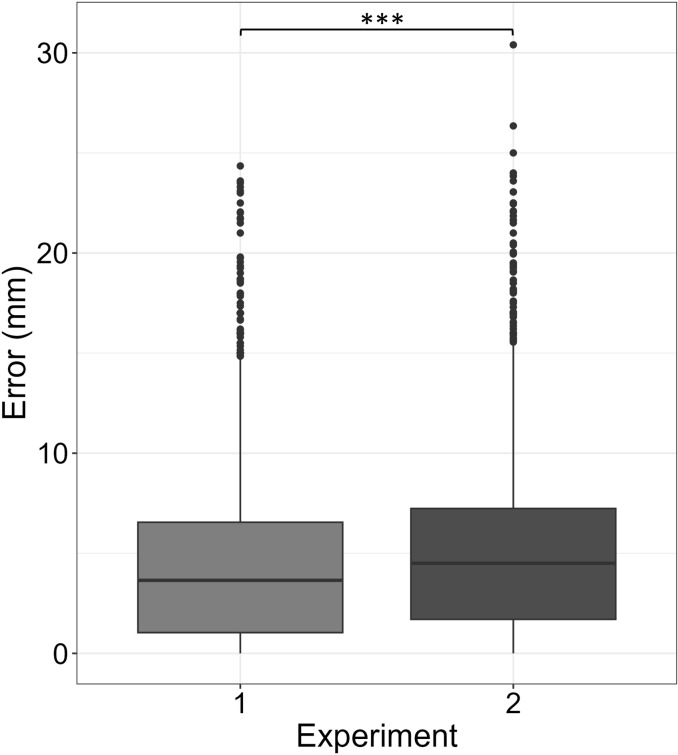
Distributions of the absolute size estimation errors of the two experiments. Boxplots represent the distributions of the errors made during in the Second Size Judgment phase in Experiment 1 (left boxplot) and Experiment 2 (right boxplot). Asterisks represent significant difference (***p < 0.001).

Similarly, we found a significant difference (*t*(4954) = −4.960, p < 0.001) in the error magnitude, with Experiment 1 showing a significant smaller error (Mean = 4.5, Std = 4.2, Median = 3.6) compared to Experiment 2 (Mean = 5.1, Std = 4.3, Median = 1.5), as well as between each interaction condition across the two experiments. The distributions of the magnitude of the errors in both experiments are represented in Fig 7. Finally, we compared the absolute errors of the First Size Judgment phase of the two experiments. This analysis aimed to assess the possible effect of the different approaches to arrive at the target location on the overall task difficulty. We found no significant difference between the two groups, nor in the raw error (p = 0.525), nor in the error magnitude (p = 0.09) of the *First Size Judgment* phase, suggesting that the different means to arrive at the target location did not affect differently the overall difficulty of the task.

## General discussion

In the current study, we aimed to assess three different aspects: i) how size perception changes after three different interaction conditions (viewing-only, reaching and grasping), ii) whether approaching the target via teleportation (Experiment 1) or via smooth locomotion (Experiment 2) could have impact on posterior size perception and iii) whether different spatial distribution of eye movements can be observed during interaction and estimation phases of the tasks.

We found that the three different types of interactions lead to different effects on the perception of the size of an object in VR. This was observed using a size judgment task where participants estimated the size of a target before and after interacting with it, alongside with approaching the target from a distance via teleportation (Experiment 1) or via smooth locomotion (Experiment 2).

Overall, size estimations after the Interaction phase showed significantly more accurate and faster responses compared to the estimations given before the interaction with the target in both experiments. This improvement can be attributed to the interaction with the target, where participants viewed the object up close, potentially allowing them to adjust their first response in the Second Size Judgment phase. This way, our manipulation may have introduced a perceptual bias, leading participants to systematically recalibrate their first response after the Interaction phase. As a result, the observed overall improvement may be due to a general test-retest effect rather than an actual enhancement in perceptual accuracy. Nevertheless, participants were significantly more accurate in reporting the target size after the Viewing-only condition compared to the Grasping one where we expected the highest improvement according to the previous literature [18,20]. This suggest that participants made significantly better estimations when they did not have to perform any hand movement on the target in both experiments. These results can be considered only partially consistent with real-world studies on the direct relationship between grasping and visual size perception [18,25,35]. Specifically, in these studies, the preparation of a grasping movement can enhance the perception of an object’s size [18] or can improve the detection of deviations in object size [25], or, after the grasping execution, there is a scale size perception by using hands’ grasping abilities as perceptual rulers [35]. In fact, this latter study suggests an interdependence of perceived sizes of objects with the action-capabilities. Linkenauger et al. found that objects looked smaller when placed in or judged relative to their right hand compared to their left [35]. They interpreted these results as demonstrating that perceivers use the extent of their hands’ grasping abilities as perceptual rulers to scale the apparent size of graspable objects. In other words, the perceived sizes of graspable objects are scaled by the action capabilities of the hand relevant to the intended action. In Bosco et al., the execution of grasping movement rescaled the size perception of two-dimensional objects following a mechanism that could be dependent on the visual and/or proprioceptive matching of the fingers with the outer border of objects [20]. In the present study, it seems that participants were not relying on the hand’s presence at all; as a matter of fact, in the informal debriefing interview, only two participants explicitly reported to have used the virtual hand grip aperture during the grasping movement as a reference when giving the size estimation in Experiment 1. Moreover, the most reported strategy to estimate the target size across both participant groups was recalling the mental image of the target. This implies that participants tried to memorize the visual representation of the target during the Interaction phase. Therefore, the presence of the hand during Grasping, might have functioned, not as facilitator, but rather as an element of interference during the memorization process, thus leading to a less accurate estimation of the target size. This aspect could be connected to the observation that most of participants tended to eventually close their index and thumb fingers more than the real dimension of the target when grasping it due to the absence of haptic feedback, as also observed in a study of Chessa and colleagues, where participants grasped objects with a virtual hand [36]. In fact, also Ozana et al. found that haptic information affects the way visual information is processed within virtual settings [37]. In particular, they found that in the absence of haptic information, grip apertures followed an atypical pattern driven by a perceptual shortcut based on relative size. However, when grasping movements included accurate haptic feedback upon contact, this relative size effect was substantially reduced across most stages of the movement, resembling patterns seen in natural 3D grasping. Hence, the smaller improvement in estimating the size of the target after the Grasping interaction in our study could be ascribed to the absence of tactile feedback, as also pointed out in Bosco and colleagues, where it was found that grasping 2D objects modulated the perception of their size, but not resulting in increased accuracy [20].

Additionally, when participants were asked to rate the difficulty of the conditions, the majority reported Viewing-only as the most difficult and Grasping as the easiest only in Experiment 2. When participants were further prompted to explain why Grasping was perceived as the easiest condition, they reported that the presence of the hand was helpful during the Interaction phase, even if they did not initially mention it, in response to the first structured question asked. This result suggests that participants may have recognized the hand’s grip aperture during the grasping action as a potential cue for the target’s size, but only when they were explicitly prompted to reflect logically on their responses. In other words, participants did not automatically use the hand’s grip as a reference for estimating the size of the object. However, when asked to consciously consider their approach to size estimation, some realized that the hand’s grip aperture could have provided information about the target’s size. This indicates that using the hand as a perceptual reference was not an instinctive behavior; rather, it required deliberate reflection to be seen as potentially useful. This finding is in line with the relevance of the task’s specific demands and the influence of its context, as illustrated in the study of Collier et al. [38]. In fact, the authors demonstrated how the scaling effect found in a study of Linkenauger and colleagues [35], where participants estimated objects as smaller depending on the intended hand to use for grasping them (predominant vs. non-predominant), likely occurred because an explicit connection between judging the graspability of the object and judging its size was provided. Therefore, since in our experiment no explicit information about the connection between grasping and the facilitation of the object’s size perception was given prior the experiment execution, participants may not have relied on the additional information that the use of the virtual hand during grasping could have potentially provided during the Interaction phase. On the other hand, most participants that took part in Experiment 1 did not report differences in the interaction conditions difficulty, but the Viewing-only condition was reported as the most helpful one to perform when evaluating the target’s size.

The direct comparison between the two experiments of this study indicates that the different means to arrive at the target location to interact with it (by teleportation or smooth locomotion) influenced the accuracy of the estimation of the target size. As a matter of fact, participants that were teleported in Experiment 1 were significantly more accurate in reporting the target’s size after the Interaction phase than participants that attended the self-motion simulation in Experiments 2. One possible explanation is the shorter time between judgments in the teleportation condition. Instant relocation may have facilitated error correction between the first and second judgments, leading to greater accuracy compared to the gradual transition in the locomotion condition. Nevertheless, this result presents an interesting contrast to prior literature suggesting that smooth locomotion—by generating optic flow linked to self-motion—should provide additional directional and distance cues that enhance perceptual accuracy [10,11]. One potential explanation for this discrepancy is that, while optic flow can indeed offer valuable spatial cues, its effects on perception in VR may differ from physical environments due to factors like sensory conflict and limited feedback. In VR, smooth locomotion often simulates movement through optic flow alone without the associated vestibular and proprioceptive feedback found in real walking. This lack of congruent sensory information can sometimes lead to disorientation, reduced spatial accuracy, or even subtle symptoms of cybersickness, which could interfere with accurate size perception. In contrast, teleportation eliminates continuous optic flow and minimizes these sensory conflicts, potentially allowing participants to focus on the size estimation task without interference from virtual self-motion. This aligns with other findings that teleportation can support spatial judgments in VR by avoiding issues linked to continuous visual flow without supporting physical motion feedback [29,39]. Thus, while smooth locomotion theoretically offers richer spatial cues, the mixed sensory input in VR may instead impair perceptual accuracy for tasks like size estimation. In fact, the employed optic flow simulation of Experiment 2 resembles joystick navigation in VR, a method that it is known to potentially compromise the VR experience compared to the more traditional teleportation locomotion technique [29]. Additional support to current results comes from previous research in VE which has demonstrated that the improvement in distance perception only comes from a physical walking action, rather than a simulated one [1].

Finally, the analysis of the fixation spatial distribution on the target during the Interaction phase of Experiment 1 revealed different results compared to real-world studies. It is known that the pattern of fixation locations on a 2D object varies between viewing and grasping tasks. During grasping movements, there is a tendency for gaze to shift towards the index finger contact point on the object, following a fixation on the centre of gravity. In contrast, during viewings tasks, all fixations tend to fall closer to the object’s centre of gravity [27]. Our findings indicate no significant differences in the distance between the first and second fixations, nor in the distance between first fixation and the centre of the target across all conditions. This suggests that, during Grasping, there was likely no gaze shift towards the upper part of the object, which participants aimed with their index finger. Furthermore, we did not find significantly different effects between the interaction conditions on the fixation locations on the comparison object in both estimation phases (First and Second), indicating that neither the preparation nor the execution of different interactions influenced the way participants looked at the comparison object. Nevertheless, participants looked significantly closer to the centre of the comparison object after interacting with it (Second Size Judgment phase). Therefore, it seems participants were not looking at the target any differently than they were looking at the comparison object. One possible explanation is that the VR environment may not evoke the same depth cues and tactile feedback present in real-world interactions, which are critical for the perceptual-motor coordination seen in natural grasping tasks. In VR, the absence of haptic feedback and the reliance on visual cues alone may cause participants to rely on a more generalized viewing strategy, focusing on the object’s center rather than specific contact points. This could indicate that, in VR, participants’ fixations are less influenced by their interaction intentions (such as grasping or reaching), leading them to treat the target and comparison objects similarly in terms of gaze distribution. This reliance on centralized fixation might also reflect a compensatory strategy to enhance size perception in the absence of detailed tactile and proprioceptive information, highlighting a perceptual adjustment unique to virtual environments.

## Conclusions

Our findings indicate that overall approaching and interacting with a target in VR, leads to faster responses and better estimations of its size. However, the interaction phase with the target may have prompted participants to rely on a more conservative, visually anchored estimate, recalibrating their judgments relative to the pre-interaction phase and not relative to the real size of the target. But interestingly, we found that performing a grasping movement towards the object does not lead to significantly more improvement compared to a reaching action or a condition where no hand movement is required, i.e., the viewing-only condition. This can suggest that, in the VR context, the interaction with the target, especially by grasping, did not enhance size perception to the same degree it might in physical environments. Moreover, we observed a deviation from the typical fixation patterns associated with grasping movements in real-world studies, suggesting that, at least in this context when relying only on visual inputs, the grasping action elicited different perceptual processes compared to real-world scenarios. Notably, different kinematic aspects of reach-to-grasp movements, such as longer movement times and larger maximum grip aperture, have already been found in VR [40]. Therefore, it would be worth exploring how grasping movement characteristics could also influence the perception of objects in our VR experiment. Finally, we observed how approaching the target via smooth locomotion led to significantly larger errors in the estimation of the target size, compared to teleportation, which is the most used way to navigate in VR. Considering this, it would be interesting to investigate whether approaching the target via a physical walking action would improve the estimation of size more than teleportation.

These investigations hold the potential to deepen our understanding of the intricate relationship between action dynamics and perceptual outcomes in virtual environments (VEs). This knowledge is particularly valuable for developing VR applications that address real-world scenarios where users need to accurately assess objects from a distance. Accurate size estimation before physical contact is essential in various contexts, such as training simulations, remote handling, and other fields requiring precise interaction with virtual objects. In the field of rehabilitation, VR offers promising avenues for designing interventions to support individuals with visuomotor impairments. By simulating different types of interactions and movements, VR can enhance perceptual accuracy and motor skills, providing targeted exercises tailored to the user’s needs. Notably, VR technology has the potential to increase patient engagement during therapy, which can help mitigate common challenges like boredom, fatigue, disinterest, and lack of cooperation that often hinder motor recovery [41]. The versatility of VR in simulating realistic tasks without physical constraints not only benefits motor rehabilitation but also has broader implications for other applications where accurate perception and interaction are critical. Understanding the specific factors that shape perception and action in VR will be essential for creating effective, adaptive therapeutic interventions, thereby enhancing the overall efficacy of VR-based rehabilitation programs.

## Supporting information

S1 Table Experiment 1: Model comparison for Model 1. (DOCX)

S2 Table Experiment 1: Full results of Model 1. (DOCX)

S3 Table Experiment 1: Full results of Model 2. (DOCX)

S4 Table Experiment 2: Full results of Model 1. (DOCX)

S5 Table Experiment 2: Full results of Model 2. (DOCX)
