# Supplementary material for: The effect of viewing-only, reaching, and grasping on size perception in virtual reality
Source: PLoS One. 2025 Jun 20;20(6):e0326377. doi: 10.1371/journal.pone.0326377 (PMC12180653; doi:10.1371/journal.pone.0326377)
Supplement: S1 Table — (DOCX) [file pone.0326377.s001.docx]

**Building of Linear Mixed Model (LMM) used for raw estimation error**

**Experiment 1**

Model comparison for **Model 1**

- Initial model

Formula: *Estimation Error ~ Size Judgment Phase + (1|Participant) + (1|Experimental Block)*

- Incorporated model

Formula: *Estimation Error ~ Size Judgment Phase + Target Size + Scale Factor + (1|Participant) + (1|Experimental Block)*

Results:

**S1 Table. Likelihood Ratio Test for model comparison.**

npar AIC BIC logLik deviance Chisq Df Pr(>Chisq)

initial model 5 30579 30612 -15285 30569

incorporated model 7 29372 29372 -14656 29313 1256.3 2 < 2.2e-16 ***

Signif. codes: ‘***’ 0.001 ‘**’ 0.01 ‘*’ 0.05 ‘.’ 0.1
