# Supplementary material for: The effect of viewing-only, reaching, and grasping on size perception in virtual reality
Source: PLoS One. 2025 Jun 20;20(6):e0326377. doi: 10.1371/journal.pone.0326377 (PMC12180653; doi:10.1371/journal.pone.0326377)
Supplement: S2 Table — (DOCX) [file pone.0326377.s002.docx]

**Full results of Model 1 analysis of Experiment 1**

**Model 1** (Incorporated model)

Formula: *Estimation Error ~ Size Judgment Phase + Target Size + Scale Factor + (1|Participant) + (1|Experimental Block)*

**S2 Table. Results of the Linear Mixed-Effects Model 1.**

| **Fixed effects** | **Estimate** | **Std. error** | **Degrees of freedom (df)** | **t value** | **p-value** |
| --- | --- | --- | --- | --- | --- |
| (Intercept) | -11.08119 | 1.46092 | 40 | -7.585 | 2.54e-09 *** |
| Second Size Judgment | -1.61344 | 0.14588 | 4788 | -11.060 | < 2e-16 *** |
| Scale factor | 16.75566 | 0.44556 | 4788 | 38.235 | < 2e-16 *** |
| Target size | -0.04714 | 0.01032 | 4788 | -4.567 | 5.07e-06 *** |

Signif. codes: ‘***’ 0.001 ‘**’ 0.01 ‘*’ 0.05 ‘.’ 0.1
