# Supplementary material for: The effect of viewing-only, reaching, and grasping on size perception in virtual reality
Source: PLoS One. 2025 Jun 20;20(6):e0326377. doi: 10.1371/journal.pone.0326377 (PMC12180653; doi:10.1371/journal.pone.0326377)
Supplement: S3 Table — (DOCX) [file pone.0326377.s003.docx]

**Full results of Model 2 analysis of Experiment 1**

**Model 2**

Formula: *Estimation Error ~ Condition * Size Judgment Phase + Target Size + Scale Factor + (1|Participant) + (1|Experimental Block)*

**S3 Table. Results of the Linear Mixed-Effects Model 2.**

| **Fixed effects** | **Estimate** | **Std. error** | **Degrees of freedom (df)** | **t value** | **p-value** |
| --- | --- | --- | --- | --- | --- |
| (Intercept) | -12.36238 | 1.46949 | 41 | -8.413 | 1.55e-10 *** |
| Viewing-only | -0.48462 | 0.25402 | 4784 | -1.908 | 0.0565 . |
| Reaching | -0.43132 | 0.25387 | 4784 | -1.699 | 0.0894 . |
| First Size Judgment | 1.42429 | 0.25739 | 4784 | 5.533 | 3.31e-08*** |
| Scale factor | 16.74848 | 0.44552 | 4784 | 37.593 | < 2e-16 *** |
| Target size | -0.04733 | 0.01032 | 4784 | -4.586 | 4.64e-06*** |
| Viewing-only*First Size Judgment | 0.26792 | 0.35918 | 4784 | 0.746 | 0.4557 |
| Reaching*First Size Judgment | 0.28930 | 0.35896 | 4784 | 0.806 | 0.4203 |

Signif. codes: ‘***’ 0.001 ‘**’ 0.01 ‘*’ 0.05 ‘.’ 0.1
