# Supplementary material for: The effect of viewing-only, reaching, and grasping on size perception in virtual reality
Source: PLoS One. 2025 Jun 20;20(6):e0326377. doi: 10.1371/journal.pone.0326377 (PMC12180653; doi:10.1371/journal.pone.0326377)
Supplement: S4 Table — (DOCX) [file pone.0326377.s004.docx]

**Full results of Model 1 analysis of Experiment 2**

**Model 1**

**S4 Table. Results of the Linear Mixed-Effects Model 1.**

| **Fixed effects** | **Estimate** | **Std. error** | **Degrees of freedom (df)** | **t value** | **p-value** |
| --- | --- | --- | --- | --- | --- |
| (Intercept) | **-9.52520** | **1.39106** | **53** | **-6.847** | **7.61e-09 ***** |
| Second Size Judgment | 0.46218 | 0.16814 | 5087 | 2.749 | 0.006 ** |
| Scale factor | 16.23884 | 0.51334 | 5087 | 31.634 | < 2e-16 *** |
| Target size | -0.06438 | 0.01194 | 5087 | -5.391 | 7.33e-08*** |

Signif. codes: ‘***’ 0.001 ‘**’ 0.01 ‘*’ 0.05 ‘.’ 0.1
