# Supplementary material for: The effect of viewing-only, reaching, and grasping on size perception in virtual reality
Source: PLoS One. 2025 Jun 20;20(6):e0326377. doi: 10.1371/journal.pone.0326377 (PMC12180653; doi:10.1371/journal.pone.0326377)
Supplement: S5 Table — (DOCX) [file pone.0326377.s005.docx]

**Full results of Model 2 analysis of Experiment 2**

**Model 2**

**S5 Table. Results of the Linear Mixed-Effects Model 2.**

| **Fixed effects** | **Estimate** | **Std. error** | **Degrees of freedom (df)** | **t value** | **p-value** |
| --- | --- | --- | --- | --- | --- |
| **(Intercept)** | **-9.12660** | **1.40317** | **54** | **-6.504** | **2.48e-08 ***** |
| Viewing-only | -0.71747 | 0.29102 | 5083 | -2.465 | 0.0137 * |
| Reaching | -0.38541 | 0.29364 | 5083 | -1.313 | 0.1894 |
| First Size Judgment | 0.30738 | 0.29550 | 5083 | 1.040 | 0.2983 |
| Scale factor | 16.23074 | 0.51309 | 5083 | 31.633 | < 2e-16 *** |
| Target size | -0.06458 | 0.01194 | 5083 | -5.410 | 6.58e-08*** |
| Viewing-only*First Size Judgment | 0.55813 | 0.41145 | 5083 | 1.357 | 0.1750 |
| Reaching*First Size Judgment | -0.11299 | 0.41519 | 5083 | -0.272 | 0.7855 |

Signif. codes: ‘***’ 0.001 ‘**’ 0.01 ‘*’ 0.05 ‘.’ 0.1
